# Displaced isolated coronal shearing fracture of the trapezoid: a case report

**DOI:** 10.1080/23320885.2021.1894941

**Published:** 2021-03-15

**Authors:** Yuya Otake, Koji Sukegawa, Kenji Onuma, Shuhei Machida, Riyo Iida, Masashi Takaso

**Affiliations:** a Department of Orthopedic Surgery, Kitasato University School of Medicine, Kanagawa, Japan; b Clinical Training Center, Kitasato University Hospital, Kanagawa, Japan

**Keywords:** Trapezoid fracture, coronal shearing fracture, isolated fracture, carpal bone, open reduction and internal fixation

## Abstract

We present a case of a displaced isolated coronal shearing trapezoid fracture, treated with open reduction and internal fixation using a headless compression screw after temporary second metacarpal-trapezoid and trapezium-trapezoid ligament detachment. Complete functional recovery was achieved. For trapezoid fractures, lateral and oblique X-ray views in supination are important.

## Introduction

Trapezoid fractures account for only 0.4% of all carpal bone fractures [1]. Anatomically, the trapezoid bone has a keystone shape, with the dorsal surface twice the size of its volar surface. It is stabilized and protected by its congruent articulations with the trapezium: the second metacarpal, the capitate, and the scaphoid. In addition, the stout second metacarpal-trapezoid ligament, trapezium-trapezoid ligament and trapezoid-capitate ligament [2] complex firmly anchors the trapezoid to these surrounding bones [3]. Hence, trapezoid fractures are rare, with 24 cases reported in the literature [4].

Isolated trapezoid fractures are exceptionally rare; to date, there are only four published reports on five isolated trapezoid fractures that were treated surgically [5–8].

We present a case of an isolated coronal shearing fracture of the left trapezoid with a dorsally displaced fragment, which was successfully treated with open reduction and internal fixation using a headless compression screw. This case elaborates the imaging findings and highlights the possible pitfalls, which can be useful for diagnosis. We present the appropriate technique and useful devices to achieve a complete reduction.

## Statement of informed consent

The patient was informed that his data would be submitted for publication, and he provided written consent. This study was approved by the Kitasato University Institutional Review Board for Observation and Epidemiological Study (approval number: KMEO B19-062).

## Case presentation

A 40-year-old man suffered a contact injury to his dominant left hand during a collision with an opponent in softball. Three days after the incident, he came to the hospital complaining of left wrist pain and swelling of the dorsum of his hand. He described an injury mechanism wherein he fell head-first and slid on the floor while extending both arms, his wrist joint flexed dorsally. His opponent exerted an axial force on his index finger during contact.

**Figure 1.**
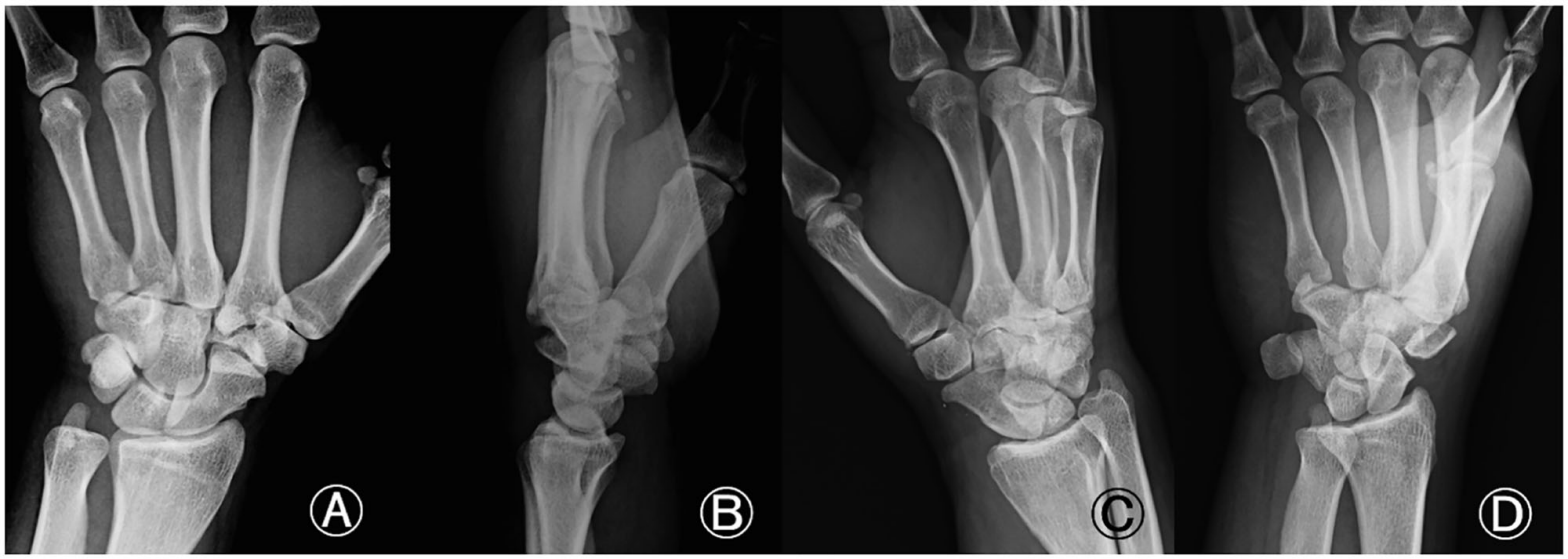
Isolated left trapezoid coronal shearing fracture in a 40-year-old male. (A) An X-ray of the wrist joint in the anterior-posterior view showing an irregular shape of the left trapezoid. (B) The lateral view in supination raised suspicion for a coronal shearing fracture of the trapezoid. (C) Oblique views recorded during pronation, showing no apparent findings. (D) The oblique view in supination clearly shows the fracture. No other carpal fractures are visible.

**Figure 2.**
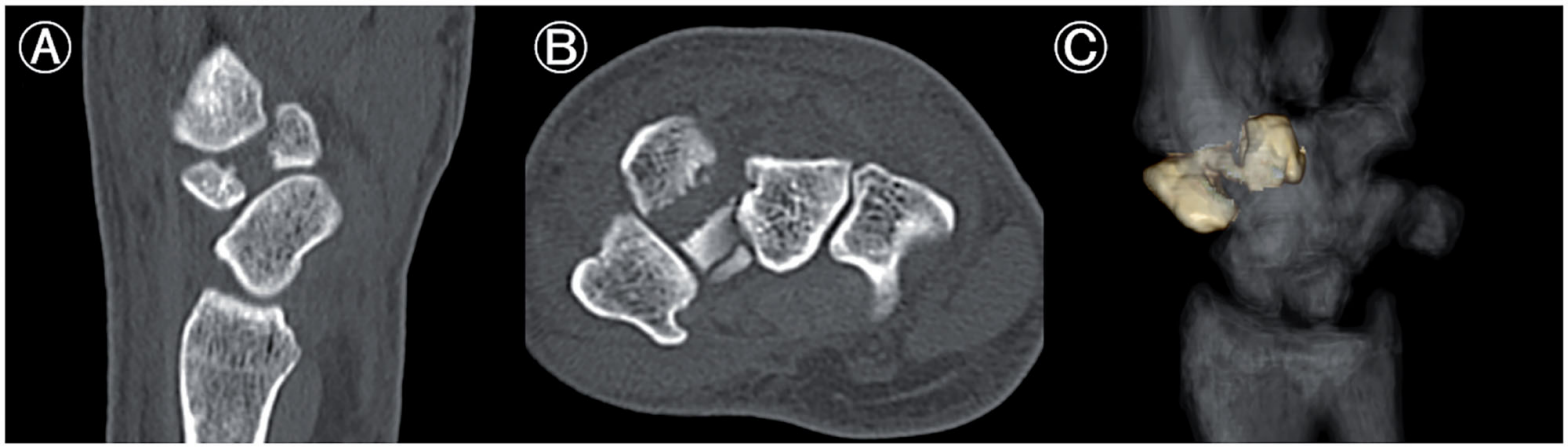
Computed tomography (CT) image of an isolated left trapezoid coronal shearing fracture in a 40-year-old male. (A) The preoperative sagittal CT image shows a coronal shear fracture at the center of the left trapezoid with a gap of 6 mm. The base of the second metacarpal bone is interposed between the fracture fragments. (B) The axial CT image shows that the palmar fragment of the trapezoid is in its original position, but the dorsal fragment is displaced dorsally. (C) On the three-dimensional CT, only the trapezoid is highlighted. A large gap and dorsal displacement of the dorsal fragment is confirmed anatomically and three-dimensionally. No abnormal positioning of the trapezoid or the other carpal bones is observed.

On examination, the left hand showed severe swelling and pain at the dorsum, and the handgrip was considerably weakened. On palpation, we found the maximum tenderness point slightly radial from the tender point of the snuff box and a subcutaneous hematoma. The X-ray of the wrist joint in the anterior-posterior view showed an irregularly shaped left trapezoid (Figure 1(A)). On viewing the lateral view in supination, we suspected a coronal shearing fracture of the trapezoid (Figure 1(B)). Oblique views recorded in pronation showed no apparent findings (Figure 1(C)). The oblique view in supination, however, clearly showed a coronal shearing fracture of the trapezoid (Figure 1(D)). No other carpal fractures were visible. The sagittal computed tomography (CT) image confirmed a coronal shear fracture approximately at the center of the trapezoid with a fracture gap of 6 mm, and the base of the second metacarpal was dislocated into the fracture site (Figure 2(A)). The axial CT image showed that the palmar trapezoid fragment was in its original position, whereas the dorsal fragment had been displaced dorsally (Figure 2(B)). On three-dimensional CT, only the trapezoid was highlighted with dorsal displacement of the dorsal fragment, and a large gap was confirmed on direct visualization (Figure 2(C)). No abnormal positioning of the other carpal bones was observed.

**Figure 3.**
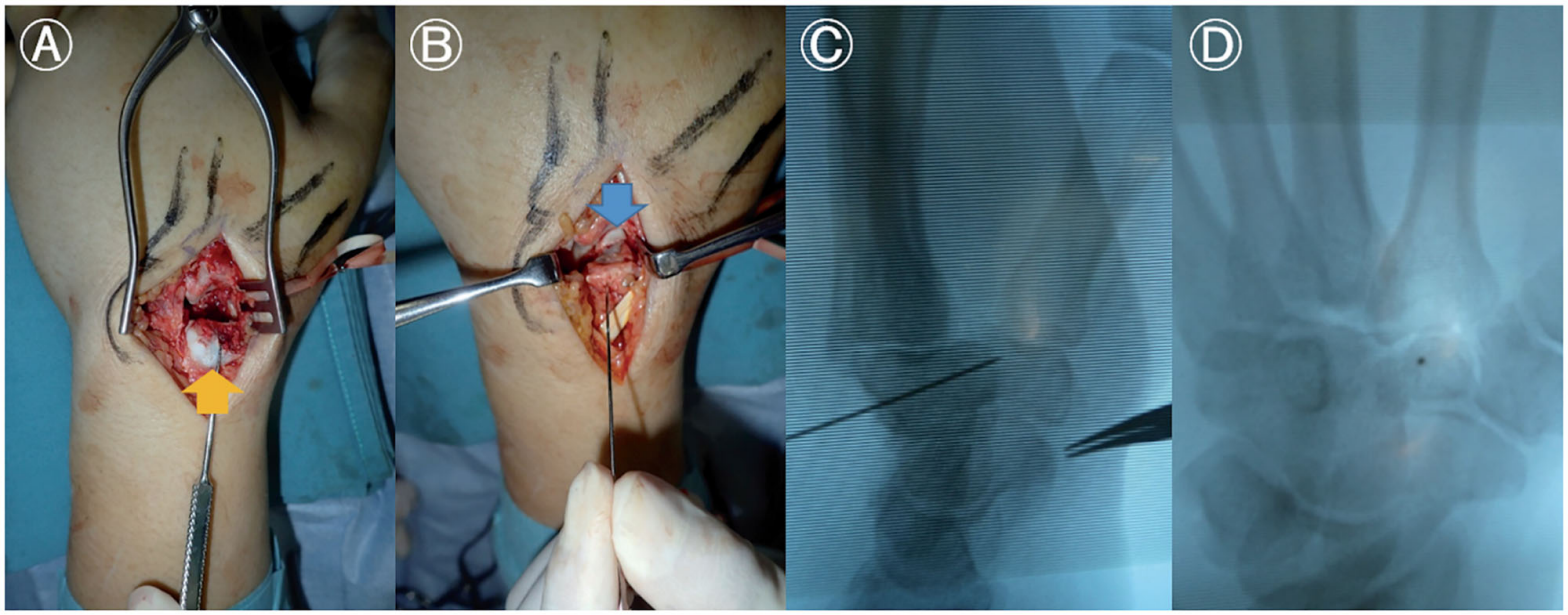
Intraoperative findings in an isolated left trapezoid coronal shearing fracture in a 40-year-old male. (A) The dorsal fragment (arrow) has been inverted to remove the hematoma, small bone fragments, and granulation tissue, that were inhibiting reduction. (B) Reduction is achieved by pushing the dorsal bone fragment toward the volar bone fragment while pulling the index and middle fingers. Guide pins are inserted, and the joint surface of the second carpometacarpal joint is inspected to ensure ideal reduction (arrow). (C) Reduction is confirmed under fluoroscopy. (D) The trajectory of the guide pin is confirmed under fluoroscopy.

**Figure 4.**
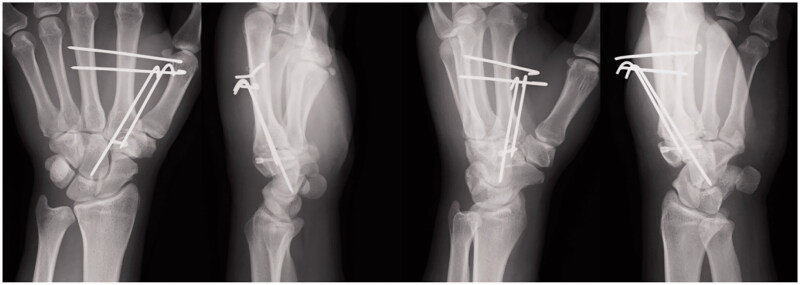
Postoperative X-ray images after surgical fixation of an isolated left trapezoid coronal shearing fracture in a 40-year-old male. The fracture was reduced and fixed with a double-threaded screw (DTJ mini screw, Meira Co., Ltd., Nagoya, Japan; width proximal 3.4 mm, distal 2.7 mm, length 20 mm). Kirschner wires were used to reduce the pressure on the trapezoid.

We scheduled an operation with a dorsal approach, four days after the injury. We made a 3 cm longitudinal incision directly over the trapezoid. The trapezoid was exposed, but the fracture site could not be identified dorsally, and reduction could not be be achieved by traction on the fingers and compression of the dorsal bone fragment. Therefore, we severed the second metacarpal-trapezoid ligament and trapezium-trapezoid ligament, and exposed the proximal surface of the second carpometacarpal (CM) joint. The dorsal fragment was identified and inverted to remove the intraarticular hematoma, small bone fragments, and granulation tissue (Figure 3(A)). Thereafter, reduction could be achieved by pushing the dorsal bone fragment to align with the volar fragment, and guide pins were inserted. We assessed the reduction by direct inspection of the proximal joint surface of the second CM joint and confirmed it under fluoroscopy (Figure 3(B,C)). The correct trajectory of the guide pin was also established under fluoroscopy (Figure 3(C,D)) before insertion of a headless compression screw (DTJ mini screw; Meira Co., Ltd., Nagoya, Japan; width proximal 3.4 mm, distal 2.7 mm, length 20 mm). After satisfactory fixation of the fracture, the dissected second metacarpal-trapezoid ligament and trapezium-trapezoid ligament and capsule were repaired. Kirschner wires were used to reduce the pressure on the trapezoid (Figure 4). Rehabilitation of the fingers was initiated immediately postoperatively. The Kirschner wires were removed after two weeks, and range-of-motion exercises were started.

**Figure 5.**
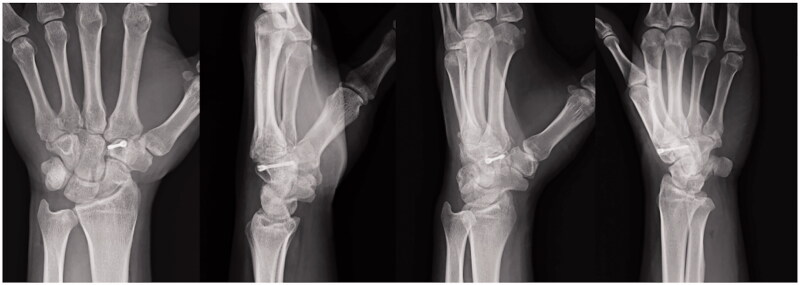
Postoperative X-ray images obtained at one year after surgical fixation of an isolated left trapezoid coronal shearing fracture in a 40-year-old male. Complete bone union and appropriate alignment of the proximal and distal carpal rows are visible. No arthritic changes of the second carpometacarpal joint or necrosis of the trapezoid can be seen.

**Figure 6.**
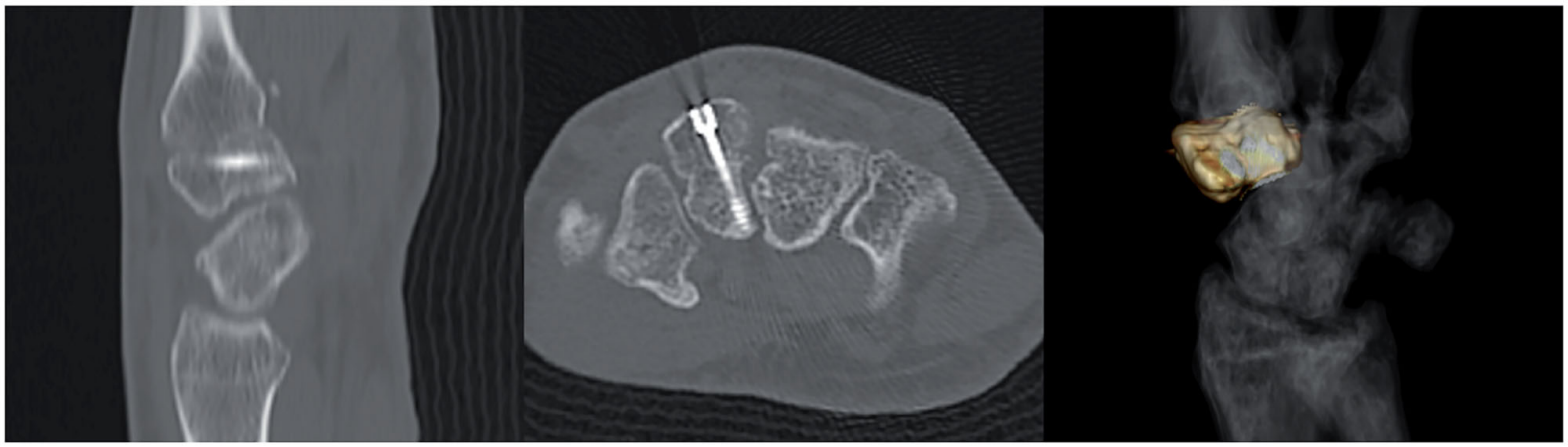
Postoperative computed tomography images obtained at one year after surgical fixation of an isolated left trapezoid coronal shearing fracture in a 40-year-old male. Complete bone union of the trapezoid is visible without any arthritic changes of the second carpometacarpal joint or necrosis of trapezoid. The headless compression screw does not protrude out of the bone. There is no step in the joint and no separation at the fracture site.

The last consultation was at one year postoperatively, and the patient denied any tenderness or pain. The grip strength was 35.1 kg, which corresponded to 90.6% of that of the right hand. The Disability of the Arm, Shoulder, and Hand score was 5, and the Modified Mayo Wrist Score was 90 points (excellent). Radiographs confirmed a complete trapezoid bone union and appropriate alignment of the proximal and distal carpal rows, with no arthritic changes of the second CM joint and no signs of avascular necrosis of the trapezoid (Figures 5 and 6). The patient returned to playing softball at the pre-injury level.

## Discussion

The mechanism of an isolated trapezoid fracture is mostly an axial or flexion force transmitted through the second metacarpal [4,9]. Miyawaki et al. [10] explained the mechanism of traumatic trapezoid injury as a sudden, excessive wrist dorsiflexion that exerts indirect stress on the trapezoid. The authors suggested that this occurs, ‘… in the same manner that a walnut is broken with nutcrackers’, illustrating the rigid fixation of the trapezoid. This may result in the dorsal displacement of the dorsal fragment because of the trapezoid’s keystone shape, with the second metacarpal acting as a lever. However, Gruson et al. [11] reported a case in which direct impact caused an isolated trapezoid fracture. In our case, the injury mechanism described by the patient appeared to be similar to previous reports.

**Figure 7.**
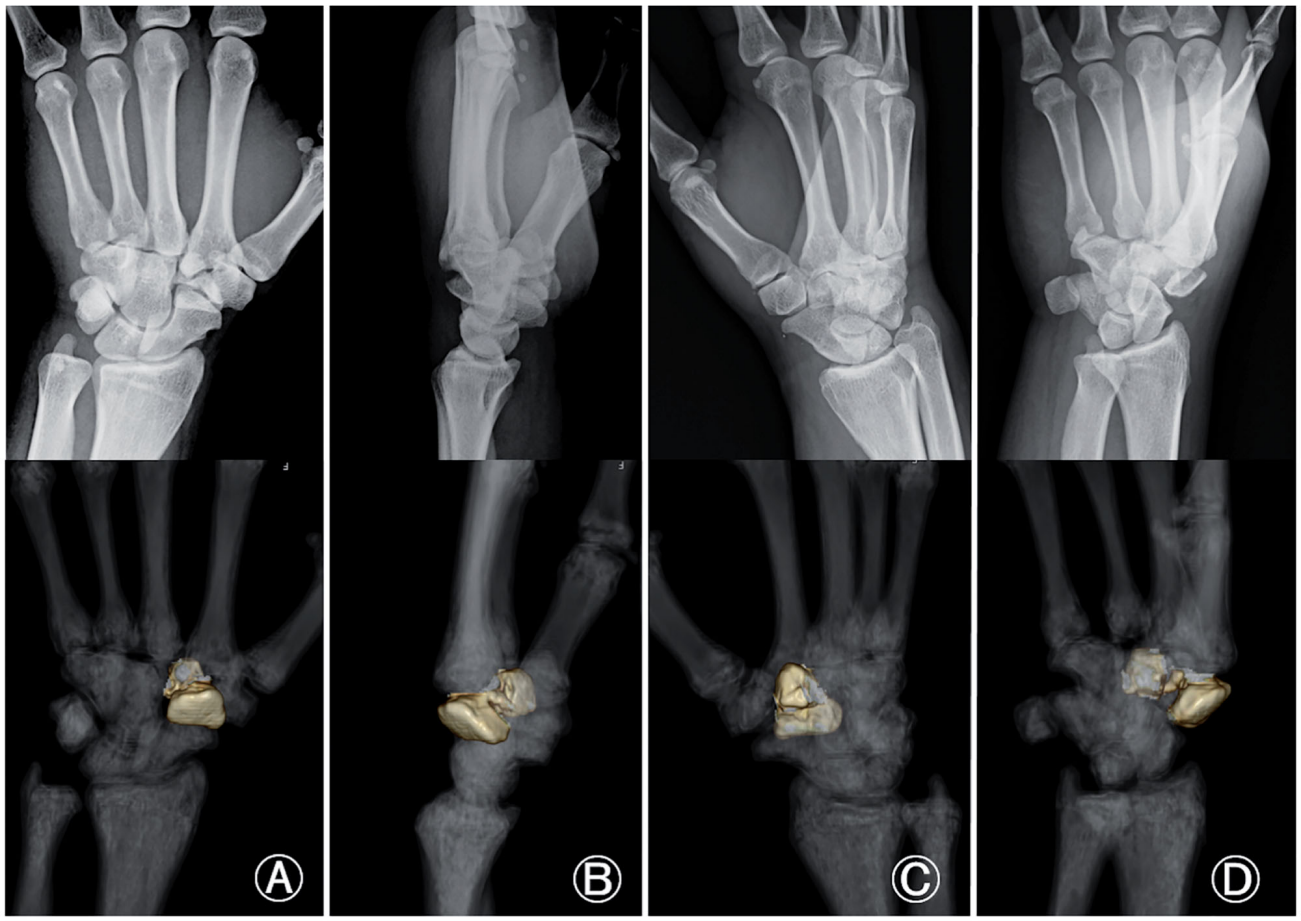
Comparison between X-ray and three-dimensional computed tomography (3 D CT) images of an isolated coronal shearing fracture of the left trapezoid. (A) X-ray in anteroposterior view and the corresponding 3 D CT; (B) X-ray in lateral view and corresponding 3 D CT; (C) X-ray in oblique view in pronation and corresponding 3 D CT; (D) X-ray in oblique view in supination and corresponding 3 D CT. The lateral and the oblique views in supination are important to identify a coronal shearing trapezoid fracture. In particular, the oblique view in supination clearly depicts a coronal shearing trapezoid fracture. No useful information could be obtained from the anteroposterior and oblique views in pronation.

Accurate diagnosis of trapezoid fractures is difficult. Kain and Heras-Palou [7] reported on 11 trapezoid fractures, including six sagittal fractures, four coronal fractures, and one crush fracture. Except for one, all the trapezoid fractures that involved a sagittal fracture were further examined with CT, magnetic resonance imaging (MRI), or both to confirm the diagnosis and determine the degree of displacement. The sagittal and crush fractures were successfully detected, but the coronal fractures could not be seen on the X-rays performed for the initial imaging assessment. Thus, the coronal fractures required a CT or MRI for diagnosis. Five of the 11 trapezoid fractures were isolated. Kain and Heras-Palou [7] highlighted the importance of further imaging when a trapezoid fracture is suspected, as an X-ray is not sufficient to rule out coronal fractures. In our case, we recognized the trapezoid abnormality on the X-ray image because of the excessive displacement of the dorsal fragment. Comparing the X-ray image and three-dimensional CT of the wrist, we noticed that careful observation of the lateral and oblique views in supination helps to identify the coronal shearing fracture (Figure 7(B,D)). In particular, the oblique view in supination depicted the coronal shearing fracture clearly (Figure 7(D)), whereas the anterior/posterior (AP) and oblique views in pronation did not provide useful information to this end (Figure 7(A,C)). This is in contrast to the fact that the oblique view in pronation is useful for identifying scaphoid fractures, which are the most common carpal fractures and are commonly encountered in daily practice. We used sagittal and axial CT images for confirming the final diagnosis, determining the indication for surgical treatment, and defining our treatment strategy.

Long-term outcomes of delayed or untreated trapezoid fractures are also unknown; however, possibilities include delayed union and non-union/mal-union and avascular necrosis [12]. For that reason, clinicians must always be accurate in their diagnosis in order to make the appropriate surgical decisions. Safran et al. [4] classified cases of trapezoid fractures with displacement of less than 1 mm as Type 1, cases with displacement of more 1 mm as Type 2A, and any adjacent fracture causing ligament compromise as Type 2B. As a treatment algorithm, the authors presented Type 2A and 2B as fractures that are surgical indications. When the algorithm was applied to 24 cases selected from previously reported trapezoid fracture papers, 89.5% of the patients received treatment in accordance with the proposed algorithm and demonstrated good outcomes. Operative treatment is suggested if there is any significant displacement (>1 mm), significant compromise of the dorsal surface, or an injury of the trapezoidal ligaments causing possible dislocation. When we have to deal with different variations of cases, we recommended excision in small fractures that do not contribute to joint stability, and consider fixation of simple fractures (not comminuted) using headless compression screws, as with other carpal bones. However, in cases of comminuted fracture which cannot be fixed with a headless compression screw, Kirschner wire fixation or external fixation is recommended; in the case of avulsion fractures, a suture anchor should be considered. In our case, the dorsal fragment was displaced by 6 mm, and complete reduction could be achieved *via* a dorsal approach, in which the second metacarpal-trapezoid ligament and trapezium-trapezoid ligament were dissected, enabling identification of the proximal joint surfaced. We believe a headless compression screw is the best device for isolated coronal shearing trapezoid fractures like our case.

## References

[1] Garcia-Elias M. Carpal bone fractures (excluding scaphoid fractures). In: Watson HK, Weinzweig J, editors. The wrist. Philadelphia (PA): Lippincott Williams and Wilkins; 2001. p. 173–186.

[2] Nanno M, Patterson RM, Viegas SF. Three-dimensional imaging of the carpal ligaments. Hand Clin. 2006;22(4):399–412. 17097462 10.1016/j.hcl.2006.08.003

[3] Suh N, Ek ET, Wolfe SW. Carpal fractures. J Hand Surg Am. 2014;39(4):785–791. 24679911 10.1016/j.jhsa.2013.10.030

[4] Safran T, Hazan J, Viezel-Mathieu A, et al. Trapezoidal fractures: overview and introduction of a novel diagnostic classification system. J Plast Reconstr Aesthet Surg. 2020;73(11):2072–2081. 32917569 10.1016/j.bjps.2020.08.069

[5] Nagumo A, Toh S, Tsubo K, et al. An occult fracture of the trapezoid bone. A case report. J Bone Joint Surg Am. 2002;84(6):1025–1027. 12063340 10.2106/00004623-200206000-00020

[6] Kam MLW, Sreedharan S, Teoh LC, et al. Severe isolated trapezoid fracture: a case report. Hand Surg. 2011;16(2):185–187. 21548157 10.1142/S0218810411005321

[7] Kain N, Heras-Palou C. Trapezoid fractures: report of 11 cases. J Hand Surg Am. 2012;37(6):1159–1162. 22522106 10.1016/j.jhsa.2012.02.046

[8] Blomquist GA, Hunt Iii TR, Lopez-Ben RR. Isolated fractures of the trapezoid as a sports injury. Skeletal Radiol. 2013;42(5):735–739. 23407926 10.1007/s00256-013-1581-z

[9] Nammour M, Desai B, Warren M, et al. Approach to isolated trapezoid fractures. Ochsner J. 2019;19(3):271–275. 31528141 10.31486/toj.18.0157 PMC6735605

[10] Miyawaki T, Kobayashi M, Matsuura S, et al. Trapezoid bone fracture. Ann Plast Surg. 2000;44(4):444–446. 10783105 10.1097/00000637-200044040-00017

[11] Gruson KI, Kaplan KM, Paksima N. Isolated trapezoid fractures: a case report with compilation of the literature. Bull NYU Hosp Jt Dis. 2008;66(1):57–60. 18333830

[12] Calfee RP, White L, Patel A, et al. Palmar dislocation of the trapezoid with coronal shearing fracture: case report. J Hand Surg Am. 2008;33(9):1482–1485. 18984327 10.1016/j.jhsa.2008.06.009

